# Time trends incidence of celiac disease in a Spanish population

**DOI:** 10.1371/journal.pone.0326634

**Published:** 2025-07-11

**Authors:** Julia María Cabo del Riego, María Jesús Núñez-Iglesias, Andrés Blanco Hortas, Tamara Álvarez Fernández, Ignacio Corchero, Silvia Novío, José Paz Carreira, Carmen García-Plata González, José Abel González-Ramirez, Sofia Zaera, Manuel Freire-Garabal Núñez

**Affiliations:** 1 Clinical Analysis Laboratory, Department of Immunology, Lucus Augusti University Hospital, Lugo, Spain; 2 SNL Laboratory, School of Medicine and Dentistry, University of Santiago de Compostela, A Coruña, Spain; 3 Department of Psychiatry, Radiology, Public Health, Nursing and Medicine, University of Santiago de Compostela, A Coruña, Spain; 4 Health Research Institute Foundation (FIDIS) of Santiago de Compostela, Lucus Augusti University Hospital, Santiago de Compostela, Spain; 5 IES Francisco Giner de los Rios. Public Secondary School, Junta de Castilla y León, Segovia, Spain; 6 Department of Hematology, Oncology Center of Galicia, A Coruña, Spain; 7 Pediatric Gastroenterology Service, Lucus Augusti University Hospital, Lugo, Spain; 8 Gastroenterology Service, Lucus Augusti University Hospital, Lugo, Spain; 9 Department of Pharmacology, Pharmacy and Pharmaceutical Technology, University of Santiago de Compostela, Santiago de Compostela, Spain; University Hospital of Bologna Sant'Orsola-Malpighi Polyclinic Department of Digestive System: Azienda Ospedaliero-Universitaria di Bologna Policlinico Sant'Orsola-Malpighi Dipartimento dell'apparato digerente, ITALY

## Abstract

**Background/Objective:**

Very few studies on celiac disease (CD) incidence across all age groups have been carried out so far, particularly in Spain. We evaluate the time trend incidence of CD of children, adults and elderly.

**Methods:**

Prospective study. Using an integrative primary and tertiary care setting approach with a standardized algorithm we identify all new cases of CD from January 1, 2012, to December 31, 2019, in a well-defined area of Galicia county, Spain. The crude incidence rate of CD was calculated as new cases per 100.000 person/year. Incidence rates were stablished by age categories 0–4, 5–19, 20–44, 45–64, 65–84, ≥ 85 and periods of 1-year intervals.

**Results:**

Between 2012 and 2019, 19,564 patients with suspicion and risk of CD were tested. 294 new cases of CD were diagnosed. Increasing CD incidence was observed from 13.11 per 100.000 person/year in 2012 to 20.92 per 100.000 100.000 person/year in 2019 (95% CI = 6.8–15.5). The temporal trend in incidence rates diverges between different age groups; showing a high incidence with stable pattern in children and young people whereas exhibiting an increasing incidence in adult/elderly, so that the incidence almost triple from 2012 to 2019. Classic CD symptoms decreasing frequency among incident cases was observed over time.

**Conclusions:**

A standardized algorithm for CD diagnosis with first-line serology testing followed by biopsy, if needed according to guidelines, confirmed the CD increasing incidence over 8 years period.

## Introduction

Celiac disease (CD) is a chronic immune-mediated disorder characterized by gastrointestinal and extra-intestinal manifestations, CD-specific autoantibodies [tissue transglutaminase 2 (TG2), endomysial (EMA) and deamidated forms of gliadin peptides antibodies], HLA-DQ2 and/or DQ8 haplotypes, and enteropathy which is developed in genetically susceptible individuals in response to gluten and related prolamins ingestion; being a gluten-free diet (GFD) an effective treatment for the majority of patients [1].

Review and meta-analysis studies showed increasing CD incidence [2–4] which varies geographically. This variation appears to be associated with several factors such as the prevalence of HLA DQ2, wheat consumption [4], age related patterns of CD presentation [4,5], race [2] as well as with testing and diagnostic paradigms [2,3,6,7]. Thus, further studies are required on the incidence of CD.

Studies are scarce on temporal trends in CD incidence, particularly including children, adults and elderly [2]. In a recent systematic review and meta-analysis [2] defining the worldwide incidence of CD (50 studies with adequate data for analysis) only 21 studies showed CD incidence across all age groups but were established with non-homogenous diagnose criteria. In this study we analyzed temporal trend in CD incidence including child, adults and elderly by using a standardized CD diagnostic algorithm, performed under control of quality [ISO, interlaboratory comparison programs for analysis and interpretation of the results (UK NEKAS SEQC), the Quality Club Control (Thermo Fisher Scientific, Uppsala, Sweden).

Furthermore, there are few studies on temporal trends in CD incidence that applied an integrative approach between different health care levels to analyze CD incidence [4,6]. In this regard, this study was based on the use of an integrative diagnostic approach between primary and tertiary health care levels. The aim of this study was to analyze the incidence of CD in a specific Galicia’s county population (Spain, Southwestern Europe) between 2012 and 2019.

## Materials and methods

### Setting

Galicia county, situated in the Southwest of Europe at the northwest side of Spain, is one of the 17 administrative regions that has his own health department.

Galicia´s Health System [Galician Healthcare Service (SERGAS)] is the major provider of health care in Galicia and provides universal and fairness of financing health care coverage for all residents. It is structured in 7 well-defined functional and organizational geographical health care areas which include coordinated health care (primary and tertiary referral university hospital per area from each one). SERGAS has a unique medical records linkage system.

This study was performed in Lucus Augusti University Hospital (HULA), a reference hospital for one of the above-mentioned well-defined geographical health areas.

In 2012, an integrative health care approach was initiated for CD diagnosis, including primary and tertiary health care providers, with the computerized standardization of the serological diagnostic procedure and an algorithm to confirm CD diagnose, according to the updated national and European guidelines [1,8].

From 2012 to 2019, 19,564 residents of HULA health-area with suspicion and/or at risk of CD were referred to HULA to rule out or confirm CD diagnosis.

The time frame used in our study (2012−2019) was determined by three main aspects: (1) ESPGHAN (European Society Pediatric Gastroenterology, Hepatology and Nutrition) 2012 CD diagnostic guidelines [1]; (2) 2020 update of ESPGHAN 2012 guide [9] and (3) to avoid Covid-19 pandemic (on 31 January 2020 was reported the first confirmed case of the 2019 novel Coronavirus in Spain).

### CD diagnosis and data collection

**Fig 1:**
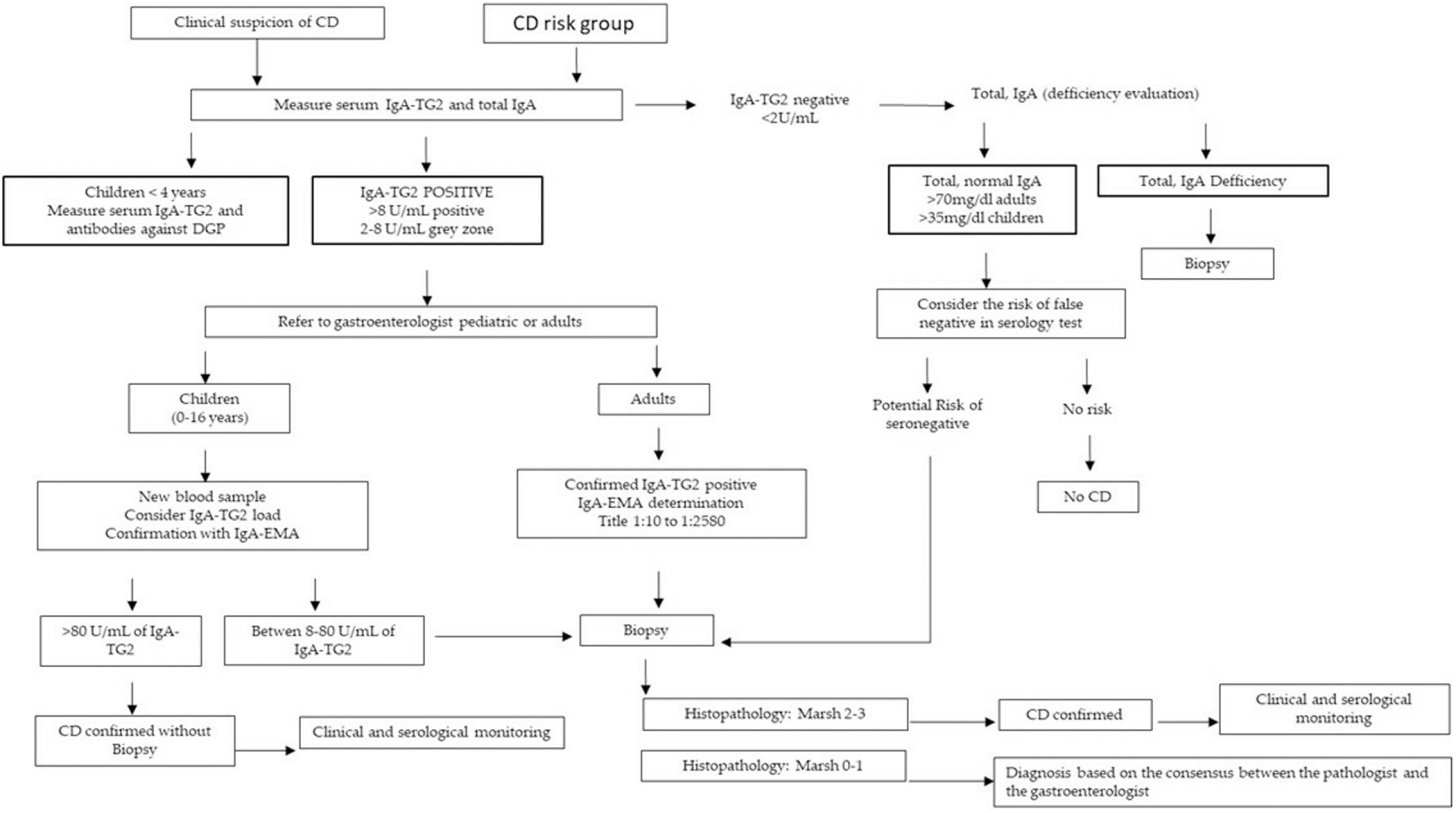
Flow chart of CD diagnosis. Abbreviations: CD, Celiac disease; IgA-EMA, Endomysial IgA antibodies; IgA TG2, Tissue transglutaminase IgA antibody.

Fig 1 and Table 1 show CD algorithm and diagnostic methods, respectively. Since serological and histopathological tests have a main role in CD diagnosis, the quality control process of those tests was guaranteed by internal and external quality assessment procedure including: (a) positive and negative control samples analyzed in each run; (b) accreditation UNE-EN-ISO 15189 standard of clinical laboratories (accreditation May 2011; reaccreditation 2013, 2015, 2017, 2019 and 2020); (c) participation in Interlaboratory Comparison Programs for analysis and interpretation of the results such as UK NEKAS (SEQC) United Kingdom and the Quality Club Control (Thermo Fisher Scientific, Uppsala, Sweden);(d) standardized biopsy and samples analysis by experts. Data was collected from 1 March to 18 December 2019, under pseudonymization techniques, and policy guidance by Institutional Ethic Committee according to current legislation.Informed consent was not required.

**Table 1:** CD diagnosis method.

	Laboratory test	Method	^*^Rf
Specific antibodies	-IgA-TG2	EliA Celikey IgA Kit Thermo Fisher Scientific, Sweden	≤ 2 U/mL positive 2,1–8 U/mL grey zone ≥ 8 U/mL positive (Biological intervals)^1^
	-IgG-DGP	EliA DGP IgG Thermo Fisher Scientific, Sweden	≥ 7 U/mL positive (manufacturer’s guidelines)
	IgG-TG2	EliA Celikey IgG Thermo Fisher Scientific, Sweden	≥ 7 U/mL positive (manufacturer’s guidelines)
	IgA-EMA	Indirect immunofluorescence AESKU-Diagnostics, Germany)	Titre <1:10
IgA Total	Total, serum IgA	Nephelometry Siemens BNII, Germany	≥ 70 mg/dL positive (manufacturer’s guidelines)
Genetics	HLA genotyping DQ2 and DQ8 haplotypes	SSPPCR Immuno and Molecular Diagnostics, Spain	Positive/negative
Histopathological Evaluation	Marsh-Oberhuber classification [10]	Evaluation Intestinal Mucosa 0,1,2, 3a,3b,3c grades.	

* Rf was established based on our population, by clinical consensus.

Abbreviations: IgA-TG2, IgA against specific tissue transglutaminase type 2 antibodies; IgG-GDP, Ig G against deamidated gliadin peptides antibodies; IgG-TG2, IgG against specific tissue transglutaminase type 2 antibodies; IgA-EMA, IgA anti-endomysial antibodies; HLA, major histocompatibility antigen; Rf, Reference value; SSPPCR, single specific primer polymerase chain reaction.

### Ethical considerations

The study follows the ethical guidelines given in the Declaration of Helsinki. This study was initiated in 2012 and concluded in 2019, following the corresponding successive approvals of Institutional Ethic Committee (last code number 2019/098). Patient´s authorization and data use were performed under operational policy and procedures established by Institutional Ethic Committee and current legislation Regulation (EU) 2016/679 and Organic Law 3/2018. Authors did not have access to information that could identify individual participants during or after data collection.

### Statistical analysis

Annual crude incidence rates were computed as new cases per 100,000 person/year all age ranges. Data from the Galician registry of statistics and the municipal register of inhabitants from 2012 to 2019 were used for the calculation of incidence. We tabulated incidence rates according to age categories 0–4, 5–19, 20–44, 45–64, 65–84, ≥ 85 and periods of 1-year intervals.

Results were summarized with absolute and relative frequencies in the case of categorical variables. Continuous variables were presented by mean and standard deviation. Comparison of categorical variables was performed with Chi-square test. The ANOVA test was used to compare means of IgA-EMA and IgA-TG2 and age groups. Smooth evolution of incidences along the years by gender and age groups were modeled by loess procedure (Local Polynomial Regression Fitting). Finally, ANCOVA (analysis of covariance) model was fitted to study the different CD manifestations. Statistically significant differences are considered with p < 0.05. SPSS 22.0 and R 3.6.3 were used.

## Results

### CD diagnosis

From 2012 to 2019, 294 new cases of CD were diagnosed. Among them, 23 (8.01%) had been identified through family screening.

**Fig 2:**
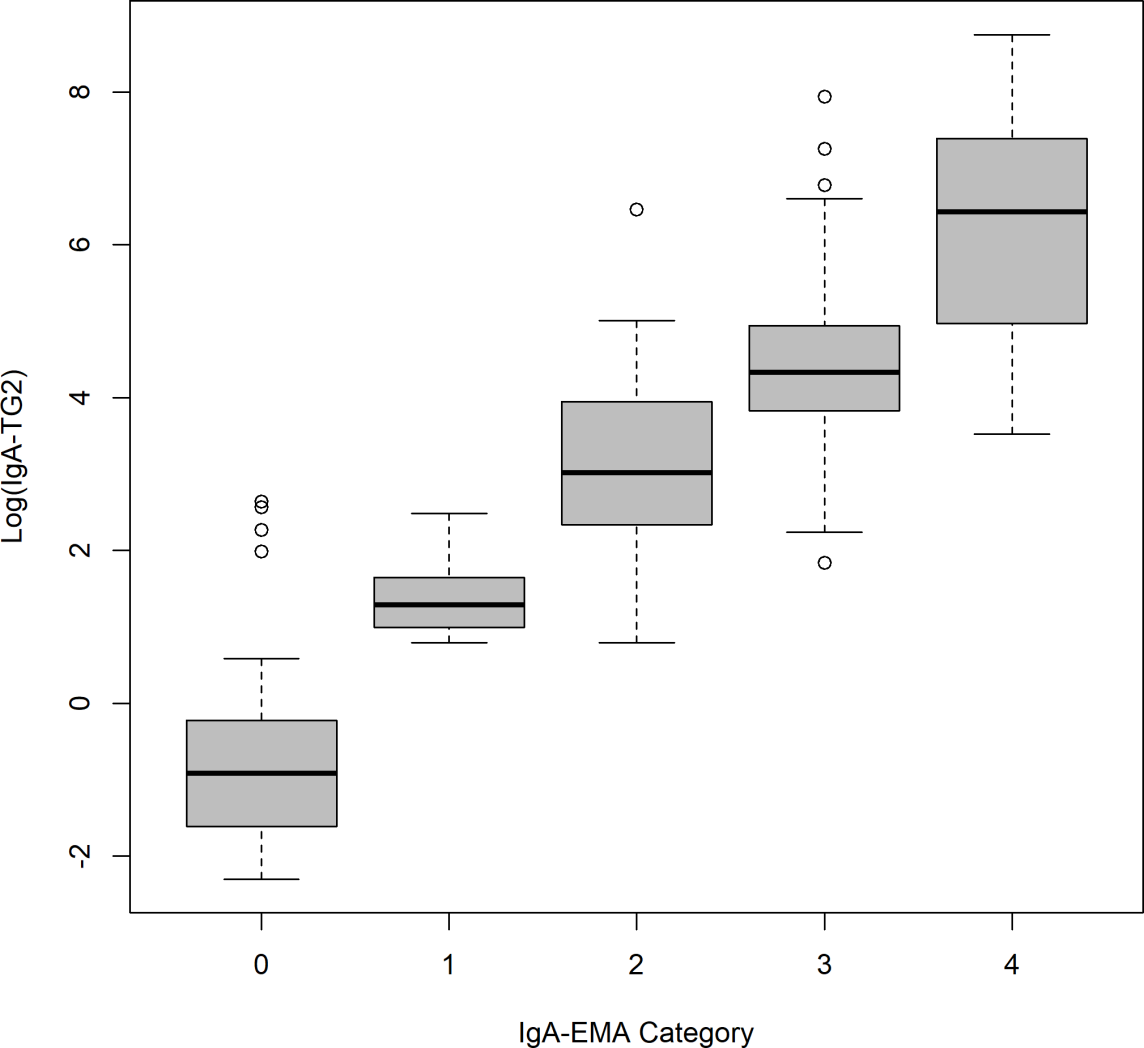
Correlation between IgA-TG2-and IgA-EMA. Abbreviations: IgA-TG2, IgA against specific tissue transglutaminase type 2 antibodies; EMA-IgA, endomysial IgA.

Table 2 and Fig 2 show results of CD serological biomarkers diagnostic test. We observed high correlation (p < 0.001) between EMA-IgA and TG2-IgA. All patients with IgA-TG2 > 2U/ml were tested for IgA-EMA and all patients with IgA-TG2 > 80U/ml showed a statistically significant relationship with the endomysium category.

**Table 2:** Values of serological diagnostic biomarkers by age groups.

Age group	TG2-IgA*	P-value	EMA-IgA category♦				
			0	1	2	3	4
0-4	620.0 (137.0–1414.0)	<0.001	2 (3.2)	0 (0.0)	2 (3.2)	16 (25.8)	42 (67.8)
5-19	102.0 (36.0–157.5)		5 (8.0)	1 (1.6)	10 (16.1)	30 (48.3)	16 (25.8)
20-44	54.5 (6.2–137.5)		23 (28.4)	4 (5.0)	11 (13.6)	23 (28.4)	20 (24.7)
45-64	43.5 (9.0–86.7)		13 (24.5)	2 (3.7)	12 (22.6)	18 (34.0)	8 (15.2)
65+	18.0 (3.0–102.2)		9 (29.0)	4 (12.9)	7 (22.5)	7 (22.5)	4 (12.9)

*Values expressed as median and interquartile range. P-value calculated from Student’s t-test. Abbreviations: TG2-IgA, Tissue transglutaminase antibody (IgA).

♦Values expressed as absolute and relative frequencies. P < 0.001. P-value calculated from Chi-square test. Abbreviations: EMA-IgA, endomysial IgA antibodies. A dilution 1:10 was considered positive and positive sera were further diluted from 1:10–1:2560.

Abbreviations: EMA-IgA, endomysial IgA antibodies. Category 0, non tested due to TG2-IgA < 2 U/mL; Category 1, EMA-IgA negative; Category 2, EMA-IgA titer 1:10–1:40; Category 3, EMA titer 1:80–320; Category 4 EMA-IgA titer 1:640–1:2560.

HLA genotyping was measured in 178 patients and their genetic distribution was: HLA DQ 2.5, 138 (77.53%); HLA DQ 8, 12 (6.74%); HLA DQ 2.2, 11 (6.18%); HLA DQ 2.5 + DQ8, 10 (5.62%); No DQ, 7 (3.93%).

The results of small-bowel biopsy are observed in Table 3.

**Table 3:** Biopsy.

Classification according to the main test used for final diagnosis	%	n
Duodenal biopsy	63.26	186
ESPGHAN diagnosis	32.31	95
Only serological and genetic diagnosis	3.74	11
**Percentage of grade of atrophy in Biopsy (Marsh grade)**	**%**	**n**
Marsh 0	1.02	3
Marsh 1	4.76	14
Marsh 2	4.42	13
Marsh 3a	11.56	34
Marsh 3b	33.33	98
Marsh 3c	7.82	23

Abbreviations: ESPGHAN, European Society for Pediatric Gastroenterology Hepatology and Nutrition.

Clinical manifestations exhibited a change in pattern from classical manifestations to extra-intestinal as well as intestinal non-classical ones (section temporal trends in CD incidence according to clinical manifestations).

### Temporal trends in CD incidence

The overall annual incidence of CD increased from 13.11 per 100.000 person/year in 2012 to 20.92 per 100.000 person/year in 2019 (Table 3). The cases with normal or minimal alteration biopsies (Marsh 0 (n = 3) and Marsh 1 (n = 14)) were diagnosed according to serological criteria (positive IgA-TG2 confirmed with IgA-EMA), compatible clinical presentation, and/or genetic risk factors. Several of these cases belonged to risk groups and had associated diseases. After differential diagnosis with other compatible conditions, CD was considered, and GLD (gluten free diet) was initiated, leading to clinical recovery and serological normalization. Cases of gluten reintroduction or transgression, the markers elevated again, and clinical alterations recurred.

The time trends of incidence rates of CD are shown in Fig 2.

Regarding age groups (Table 4), children´s incidence was high over the entire study period in comparison to the remaining age groups (Table 4, Fig 2 A and C). Particularly, high incidence rate was observed in female children <4 years of age. In adults/elderly age groups the annual incidence rates increased over the study period. Thus, the incidence rate was 6.79 per 100,000 person/year in 2012 and 16.55 per 100,000 person/year in 2019. Specifically, in women aged 45–64 and 65–84 years, significant increase in incidence were seen compared to men in the same age ranges (Table 5; Fig 2B).

**Table 4:** CD incidence per 100.000 person/year (median ± CI 95%).

Year	Total	Children/ Young	Adult/ Elderly
2012	13.11 (8.33–17.88)	53.69 (27.38–79.99)	6.79 (3.09–10.48)
2013	18.66 (12.94–24.37)	51.13 (25.62–76.99)	13.68 (8.423–18.93)
2014	18.36 (12.67–24.04)	61.46 (33.07–89.84)	11.67 (6.79–16.54)
2015	13.89 (8.91–18.86)	37.86 (15.49–60.22)	10.17 (5.59–14.74)
2016	15.39 (10.13–20.64)	55.14 (28.12–82.15)	9.17 (4.81–13.52)
2017	18.35 (12.59–24.10)	34.53 (13.13–55.92)	15.79 (10.04–21.53)
2018	17.52 (11.87–23.61)	48.27 (22.99–73.54)	12.63 (7.46–17.79)
2019	20.92 (14.73–27.10)	48.20 (22.95–73.44)	16.55 (10.62–22.47)

**Table 5:** CD incidence by gender and age group per 100.000 person/year.

Age Group	2012-2013	2014-2015	2016-2017	2018-2019
**Female**				
0-4	109.62 (33.69–185.54)	140.02 (53.29–226.74)	127.26 (44.16–210.35)	129.35 (44.89- 213.80)
5-19	41.33 (14.33–68.32)	14.05 (0.81–29.94)	33.04 (8.56–75.51)	51.33 (21.00–81.65)
20-44	20.14 (9.59–30.68)	16.65 (6.81–26.58)	23.97 (11.84–36.09)	25.26 (12.47–38.04)
45-64	8.4 (1.03–15.76)	13.17 (4.04–22.29)	12.91 (3.96–21.85)	20.63 (9.41–31.84)
65-84	9.2 (1.13–17.26)	9.46 (1.16–17.75)	7.82 (0.15–15.48)	16.07 (4.93–27.20)
≥85	6.92 (0.16–20.48)	0.0 (0.0–0.0)	0.0 (0.0–0.0)	5.9 (0.24–17.46)
**Male**				
0-4	119.08 (41.32–196.83)	94.43 (24.50–164.35)	108.62 (33.39–183.84)	42.16 (0.02–89.86)
5-19	30.64 (7.94–53.33)	44.58 (16.95–72.20)	31.32 (8.12–54.51)	39.92 (13.84–64.99)
20-44	10.02 (2.59–17.44)	7.55 (0.93–14.16)	12.75 (3.91–21.58)	8.37 (1.03–15.70)
45-64	3.24 (0.63–7.72)	6.43 (0.12–12.73)	8.01 (0.99–15.02)	9.59 (0.91–17.26)
65-84	4.55 (0.75–10.85)	9.25 (0.18–18.31)	4.67 (0.08–11.14)	2.35 (0.12–6.95)
≥85	12.46 (0.01–36.87)	11.5 (0.01–34.04)	0.0 (0.0–0.0)	0.0 (0.0–0.0)

Data expressed as median ± CI 95%.

When considering gender, divergent time trends in CD were found. Among women, incidence increased from 2016 to 2019 whereas among men, incidence has been in decline since 2016 (Fig 2B).

### Temporal trends in CD incidence according to clinical manifestations

**Fig 3:**
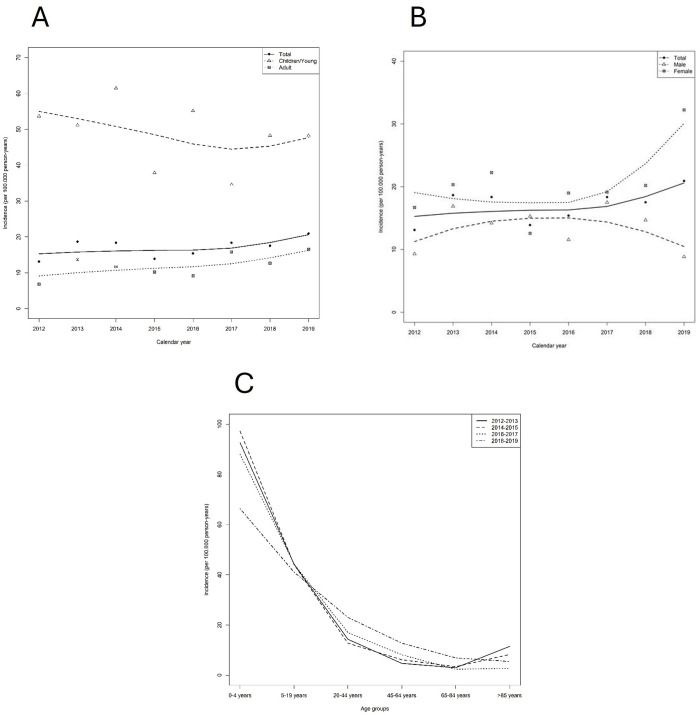
Rates of CD incidences along time adjusted by Loess Smoothing Methods. **(A)** By age group. **(B)** By gender. **(C)** Age trends of CD incidence by groups of 2 natural years adjusted by Loess Smoothing Method. Locally estimated scatterplot smoothing with 95% confidence intervals (dashed lines).

**Fig 4:**
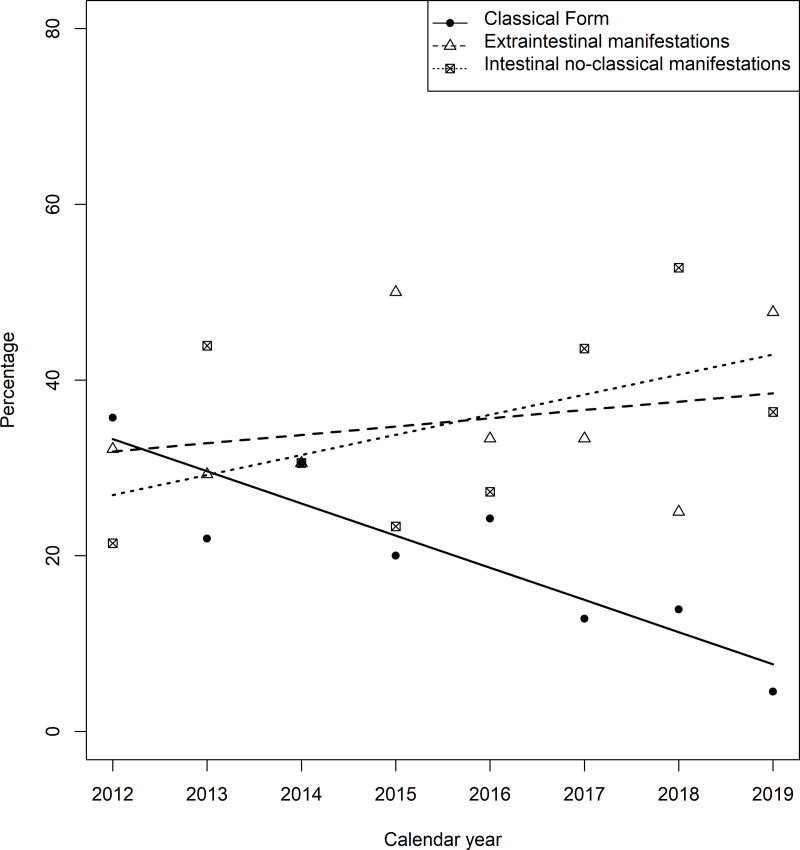
Temporal tends in the proportion of classical manifestations in incidence CD cases between 2012 to 2019. ANCOVA regression lines. Abbreviations: ANCOVA, analysis of covariance; CD, celiac disease.

Fig 3 shows temporal tend in the proportion of CD patients with clinical manifestations. Over time the proportion of cases showing classic manifestations (diarrhea, weight lost, etc) decreased significantly (p < 0.001) while extra-intestinal, increased (anemia, osteoporosis, infertility, etc.) as well as intestinal non classical ones (abdominal distension, esophagitis, etc.) (Fig 4).

## Discussion

This study is the first Spanish study on time trend of CD incidence that includes children, adults and elderly in Spanish population. Other studies in our country are specifically targeted at children [11,12] or adults [13].

Throughout this discussion, we will contrast the main findings with those from other studies, considering methodological aspects, characteristics of the sample and other aspects that could influence the results.

We have verified an increasing CD incidence from 13.11 per 100.000 person/year in 2012 to 20.92 per 100.000 person/year in 2019. Comparing this data with those corresponding to other countries [2,4,6], reveals similar evolution. For example, Italy exhibited <4.6 per 100.000 person/year and >12.7 per 100.000 person/year before and from 2000 onwards. Nevertheless, our reported incidence is higher.

Those studies that cover approximately the last half of the 20th and the first years of the 21th century [2,14] provide discordant intra and intercountry variability. Probably due to the different aspects such as diagnostic algorithm used, coordination between the different levels of health care for the diagnosis of CD, data source for incidence calculation, screening strategies to identify CD, as well as race. In addition, other aspects can influence such as diet, gluten related factors, etc.

The number and quality of available serological and genetic tests can be confusing factors [15]. Particularly, a large variation in CD diagnostic accuracy was observed [15]. A Spanish study [16] enrolling Spanish Clinical Laboratories from the 17 administrative regions (each with their own public health care system) showed high variability in the use of serological markers of CD in primary care. To the best of our knowledge, this is the first study both in Spain and in southwestern Europe to use an algorithm in a wide sample of patients, −19,565 patients- of all ages from 0 to 87 years. We used TG2-IgA for the first-line screening. IgA-EMA test was performed in positive or borderline samples to increase positive predictive value (accreditation UNE 15189 and use the same observed for IgA-EMA). In the pediatric groups of age, intestinal biopsy was avoided according to our algorithm based on the ESPGHAN guidelines [1] when the child exhibited typical symptoms and signs of CD as well as high titters of IgA-TG2, detectable endomysial antibody, and HLA-DQ2/HLA DQ8 positivity. Nevertheless, these criteria are not used worldwide, where biopsy is still required largely in pediatric and adult cases to establish CD diagnosis.

Another aspect to consider is that CD screening strategies vary depending on the country and even the geographic area [2,3,6,13,15,17,18] and CD screening recommendations do not always achieve unanimity [4,9,19–22].

We should mention that for CD screening, and specifically on first-degree relatives, our practice is aligned with ESPGHAN guidelines [1,9], the Spanish Working Group on CD diagnosis [8] and NICE [320]. Thus, we identified 8.0% of new CD cases from first-degree relatives of CD patients. It is to note the percentage magnitude. Other studies on children from high-risk families (Leiden, the Netherlands) [23] based on predictive model) showed a cumulative incidence from 7.5% to 17.5% at 3 and 10 years of age, respectively. In Spain, a recent study [18] has verified that the screening of relatives of people with CD must be improved since family screening has not been recommended in 20% and 40% of pediatric and adult cases with CD, respectively.

If we considered gender, we verified higher CD incidence in women, particularly among adult/elderly group versus children and young people (54.1% vs. 67.4%). We attributed this finding, at least in part, to more medical testing and higher prevalence of anemia which orients to CD testing [24]. Similarly, increasing trends in female CD rates have been also reported in other countries [5]. Some studies showed that the risk of undetected CD appeared to be higher among females compared to males (RR, 1.42; 95% CI, 1.27–1.57; P < .00001) [25]. Nevertheless, it was showed that when men show diarrhea or weight loss is less likely to undergo duodenal biopsy and consequently there could be underdiagnosed [26].

CD has high prevalence in the Nordic countries or some zones of North Africa where there is a high proportion of HLA-DQ2.5 and/or DQ8 carriers. In Western Europe, the Middle East and North Africa, CD is common in paralleled to HLA-DQ2.5 and DQ8 that reach 20% and 10% [4,27]. In our Health Sanitary Area, we observed similar Haplotypes distribution [28,29]. Paraphrasing to Iversen and Sollid [30], HLA-DQ25 is considered a “chief factor” which is exhibited by around 90% of patients, showing an equal distribution between HLA-DQ2.2 and HLA-DQ8 remaining patients. Nevertheless, HLA genes are a necessary but not a sufficient factor to develop CD. Thus, only 3% of all European individuals harboring HLA-DQ2.5- or HLA-DQ8 loci that have gluten consumption develop CD [31]. In the same way, among CD patients in Spain 3% are negative for HLA-DQ2.5 and HLA-DQ8. Non-HLA risk loci could be involved in CD pathogenesis [32,33].

Regardless of the points aforementioned, other factors, which will be the subject of our future investigations, could be a contributing factor. Bread is the most common wheat-based food in Spain [34] and Galician county, where our study was conducted, is the geographical area of Spain with the highest consumption of bread [35], even compared to other countries, such as US [36] or Italy [37], where its consumption is very common. Also other gluten-related factors (vital gluten as a food additive, variations in individual diets with regard to the amount and types of wheat consumed, wheat genetics, and agronomic practices (such as nitrogen fertilization) could contribute to determining the effect of wheat [38–42] for people with the appropriate genetic susceptibility for CD; mainly those carrying the genes for particular proteins of the major histocompatibility complex, DQ2 and DQ8. Therefore, further research would be needed to evaluate such factors. Also, breastfeeding could be a potential protective factor [10,43–45]. In Spain breastfeeding is increasing [46]. Additionally, other environmental factors such as gastroenteritis or other infections could influence CD risk. As an example, H. pylori infection is associated to CD risk [43] and its seroprevalence among Spanish adult population is high [44].

The main limitation of the present study is that population is predominantly Caucasian. Thus, our data could not be extrapolated to other populations. The main strength of our study is an integrative health care approach with the availability of unique applicable algorithms under accreditation quality criteria. In our study there was used a standard computerized algorithm used by the physicians and pediatrics of primary care as well as hospital pediatricians and gastroenterologists of a well-defined health area (see Methods section). Murray et al. [5] or Ludvigson et al. [6] were precursors in postulating an integrative approach between health care providers.

## Conclusions

A increase in incidence of CD between 2012 and 2020 has been observed. Standardized algorithm for CD diagnosis with first-line serology testing followed by biopsy, if needed, confirmed the CD incidence increase over 8 years period.
